# Laboratory Evaluation of Flurox, a Chitin Synthesis Inhibitor, on the Termite, *Microcerotermes diversus*

**DOI:** 10.1673/031.010.0201

**Published:** 2010-02-18

**Authors:** Behzad Habibpour

**Affiliations:** Department of Entomology, College of Agriculture, Shahid- Chamran University, Ahwaz, Iran

**Keywords:** subterranean termite control, insect growth regulators, termite bait, molting disruption

## Abstract

*Microcerotermes diversus* (Silvestri) (Isoptera: Termitidae) is the most economically destructive termite in structures in southwest Iran. One sustainable control strategy that usually helps to reduce subterranean termite damage in buildings, is the use of insect growth regualtors in a suitable bait matrix that are safe to the user and the environment. In the laboratory assays described here, the delayed toxicity of Flurox, a chitin synthesis inhibitor, to *M. diversus* was evaluated under force-feeding and choice trials. Flurox induced worker and nymph mortality and incomplete ecdysis in nymphs of *M. diversus* under no-choice and two-choice feeding tests. These adverse effects may cause disruption of the caste balance in *M. diversus*, leading to the collapse of the colony. These assays determined concentrations of Flurox that can be used in bait formulations.

## Introduction

During recent years an extensive survey on the foraging behavior of the most damaging subterranean termites of Khuzestan province (Iran) has been undertaken with a view to the development of appropriate strategies for control. Four genera namely *Microcerotermes, Amitermes* (Termitidae), *Anacanthotermes* (Hodotermitidae) and *Pssamotermes* (Rhinotermitidae) were the main genera collected in Iran. Current management of subterranean termites in Iran mainly involves the application of a soil insecticide to reduce/isolate their foraging populations (Habibpour 2006). Organochlorine insecticides are still used in Iran to control subterranean termites. These compounds have undesirable environmental effects.

*Microcerotermes diversus* (Silvestri) (Isoptera: Termitidae) is an extremely destructive structural wood pest, and is considered to be the major species with a wide distribution in Iran, Iraq and Oman (Edwards and Mill 1986). *M. diversus* was identified as the major pest of date palms (*Phoenix dactylifera* L.) in Iran, Iraq and Saudi Arabia (Logan and El Bakri 1990). Its nest is very complicated, diffuse and cryptic. The developmental pathway of the genus *Microcerotermes* has, as in all Termitidae, an irreversible bifurcation at the first molt, separating the nymphal/alate line from the worker / soldier line. Small workers are male, large workers are female, and soldiers develop from them. Workers are capable of developing into presoldiers at the first instar (Roisin 1990). This species tends to form secondary nests containing both reproductives and brood. In locations where the ground water is high, secondary nests are usually built in above-ground sites such as tree trunks and wall voids.

Soil treatments with organochlorines, organophosphates and carbamates do not persist for long in this environment and proved ineffective against aerial colonies. In other parts of the world insecticidal baits have been shown to be an effective alternative to conventional soil insecticides for remedial termite control (Su 1991). Bait systems can eliminate entire colonies of subterranean termites (Grace et al. 1996; Su and Scheffrahn 1996). Methods for the control of termites including chemical control, baiting system and wood protection, have hardly been investigated scientifically in Iran.

Cellulose baits impregnated with insect growth regulators can be used for termite control (Su et al. 1985; Jones 1984, 1989; Jones 1984, 1989; Haverty et al. 1989; Su and Scheffrahn 1993; Su 1994). Many insect growth regulators induce development of superfluous presoldiers and soldiers, cause morphological abnormalities (intercastes), defaunation of cellulose digesting microbes, and exert various levels of acute and delayed toxicity. However, termite responses to insect growth regulators are not uniform (Jones and Lenz 1996). Most laboratory and field experiments with insect growth regulators so far have focused on the lower termites (Rhinotermitidae) and higher termites have been studied only rarely. For example, the efficacy of insect growth regulators to control the genus *Microcerotermes* spp. was studied in the field by Faragalla et al. (1985) and laboratory efficacy of flufenoxuron 10 DC on mortality of *Microtermes obesi* (Isoptera: Termitidae) in soils was evaluated by Ahmed and Farhan (2006) in Pakistan, but their results were inconclusive.

The objective of this study was to determine the general effects of the chitin synthesis inhibitor Flurox (flufenoxuron) on *M. diversus* including mortality and molting inhibition and differences in response between castes.

## Materials and Methods

In Khuzestan province, in the period of 2006– 2007, termites (workers, nymphal 3^rd^ to 5^th^ instars and soldiers) were collected from a field colony of *M. diversus* in the campus of Shahid-Chamran University (without exposure to pesticides) using a collection trap unit as described by Sornnuwat et al. (1996) with some modifications. The unit was made of PVC pipe with a roll of toilet paper and a beech wood (*Fagus orientalis*) stake in it, buried in the soil. The chitin synthesis inhibitor tested was Flurox (common name: flufenoxuron), provided by Janssen Pharmaceutica, Belgium.

### Laboratory no choice trial

Flurox was dissolved in methanol (Merck, www.merck.com). Four concentrations (100, 500, 1000 and 5000 ppm) were prepared. Whatman No. 1 filter papers (9.0 cm in diameter) were uniformly wetted with 1 ml of each concentration or with 1 ml of methanol as a control. After overnight air drying each filter paper was then placed in a Petri dish (10 cm in diameter) and 1 ml of distilled water added to moisten the paper. One hundred workers, 30 nymphs and 2 soldiers were introduced into each dish. The soldiers were added to reflect natural caste ratios and prevent molting of workers to soldiers. Four replicates were prepared for each concentration. Experimental units (Petri dishes) were kept in constant darkness at 28 ± 1° C and 90 ± 5% relative humidity in an incubator. The number of dead workers and dead nymphs (caused by molting inhibition or direct toxicity) were counted daily for each treatment for 28 days after treatment. Dead workers were counted and removed daily from the test unit (Remmen and Su 2005). Data were normalized by

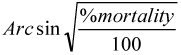

and then subjected to a one-way ANOVA (SPSS 16). Significant means were separated by the Tukey multiple range test (*P* < 0.05), however, untransformed means are reported. In tables mortality was corrected by Abbott's formula.

### Laboratory choice trial

Three concentrations of Flurox (500, 1000 and 5000 ppm) were prepared as above. Two filter papers (4.25 cm in diameter) were placed in each Petri dish (10 cm in diameter), and they were treated with 0.5 ml of one of the concentrations of Flurox or methanol alone as a control, and allowed to air dry. There was a space (1 cm) between the papers. Other conditions were similar to that of the no-choice tests. Analysis was the same as above.

## Results

### Laboratory no-choice trial

**Figure 1:**
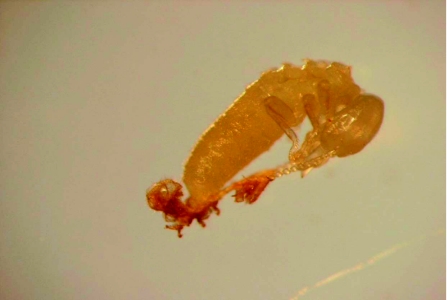
Adverse effect of Flurox on nymphs of *Microcerotermes* *diversus.* Death of nymph due to molting failure. High quality figures are available online.

The mortality of workers and nymphs in all control groups was always lower than 4%. All concentrations of Flurox greater than 100 ppm elicited 100% mortality of the workers (Table 1). Significant differences were observed among the concentrations throughout the experimental period (Table 1). In all concentrations (with the exception of the control groups) nymphs died due to direct toxicity or during metamorphosis when nymphs showed symptoms of molting failure after exposure to Flurox, Figure 1. The largest number of dead nymphs was observed at concentrations of 1000 and 5000 ppm (Table 1).

**Table 1:**
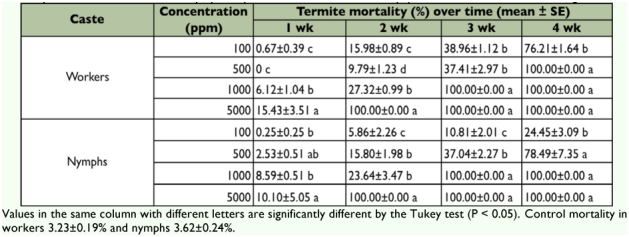
Mortality of *M. diversus* workers and nymphs exposed to Flurox treated filter paper disks in the no-choice feeding test.

### Laboratory choice trial

In choice tests, Flurox treatment at 500 and 1000 ppm worker mortality varied from 32.5%, to 100% (Table 2). The percentages of nymph mortality due to direct toxicity or during metamorphosis (molting failure) varied from 53%, to 100% (Table 2). No difference in feeding behavior was noted at a concentration of 5000 ppm; they did not appear to be repelled by the presence of Flurox. Flurox was found to have relatively low (< 21%) contact toxicity, so the toxic effect was mainly due to consumption (Table 3). The nymphs directly fed on the filter paper disks as they died faster without workers present (compare Tables 2 and 4).

**Table 2:**
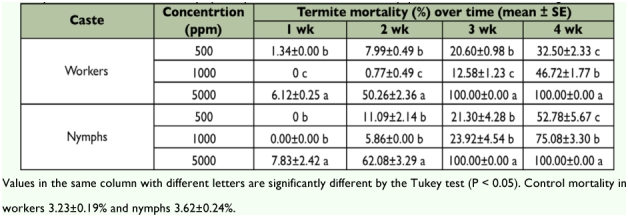
Mortality of *M. diversus* workers and nymphs exposed to Flurox treated filter paper disks in the choice feeding test.

**Table 3:**
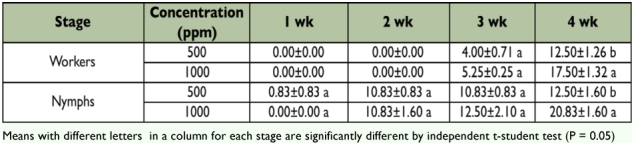
Contact mortality (% ± SE) of *M. diversus* workers and nymphs exposed to flurox

**Table 4:**
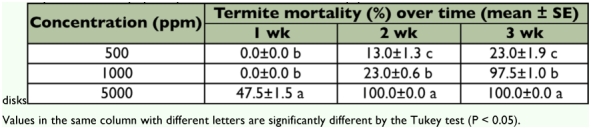
Mortality of *M. diversus* nymphs exposed to the flurox treated filter paper

## Discussion

The development of alternative strategies to control multigenera/multispecies termite infestations that avoid pesticide accumulation in soil is an important goal. One of the commonly used strategies of integrated termite management is the use of baiting systems. Several novel slow-acting pesticides are being screened for the control of subterranean termites (Hrdy et al. 2004; Rojas et al. 2004; Kubota et al. 2006; Yeoh and Lee 2006) because use of slow-acting insecticides in baiting systems can be distributed within, and kill, the entire colony. This is the first report of using Flurox as oral bait toxicant to control the genus *Microcerotermes.*

Concentrations of Flurox did not produce high mortality in *M. diversus* workers and nymphs at the first week, although this mortality was higher in no choice treatments. The results clearly show that Flurox is a slow acting toxicant, which is an important characteristic for use in baits. This compound could be distributed among termites in the field colonies when applied in bait stations. This agrees with other studies that showed delayed toxicity for chitin synthesis inhibitors (Su and Scheffrahn 1993; Lenz et al. 1996; Kubota et al. 2006).

Flurox treatments against *M. diversus* in no-choice and choice feeding tests produced similar results at concentration of 5000 ppm. Flurox is a potent chitin synthesis inhibitor to control wood pests. Flurox has been found to be extremely active against wood borers, when applied in preventive or curative way (Valcke and Pallaske 1995). During the reevaluation of molt-inhibitors it was found that Flurox could be an appropriate replacement for the pyrethroids in wood preservation (Pallaske 1997).

Control of termites with a baiting method would be successful if the foraging behavior is properly understood. Laboratory evaluations, using groups of older workers and soldiers, can fail to accurately predict the impact of the insect growth regulators at the level of greatest concern, the colony (Lenz et al. 1996). Usually, in *M. diversus* winged nymphal instars 3–5 were observed in feeding sites. They were found in the galleries leading to different food sources, including cellulose baits and monitoring stakes near the colonies in the summer (Habibpour 2006). To simulate the conditions of field treatment, the nymphal instars were used in laboratory trials.

The results presented here show that concentrations of 1000 and 5000 ppm of Flurox have the potential to be used in termite baiting. The results suggest that the caste system and foraging behavior may be the most important factors affecting the success of termite control with baiting. We have an ongoing field program aiming to develop effective, cheap bait formulations with locally occurring bait toxicants and bait matrices for the control of termites in Iran.
